# Can oral bacteria affect the microbiome of the gut?

**DOI:** 10.1080/20002297.2019.1586422

**Published:** 2019-03-18

**Authors:** Ingar Olsen, Kazuhisa Yamazaki

**Affiliations:** a Department of Oral Biology, Faculty of Dentistry, University of Oslo, Oslo, Norway; b Research Unit for Oral-Systemic Connection, Division of Oral Science for Health Promotion, Niigata University Graduate School of Medical and Dental Sciences, Niigata, Japan

**Keywords:** Oral and intestinal dysbiosis, dissemination of oral and intestinal bacteria, gut, animal and human studies, periodontitis intervention

## Abstract

Oral bacteria spreading through the body have been associated with a number of systemic diseases. The gut is no exception. Studies in animals and man have indicated that oral bacteria can translocate to the gut and change its microbiota and possibly immune defense. The ectopic displacement of oral bacteria particularly occurs in severe systemic diseases, but also in patients with “chronic” periodontitis. Thus, *Porphyromonas gingivalis*, which creates dysbiosis in the subgingival microbiota and immune defense, may also cause dysregulation in the gut. A dysbiotic gut microbiota may cause diseases elsewhere in the body. The fact that “chronic” periodontitis may affect the gut microbiota could imply that consideration might in the future be given to a coordinated approach to the treatment of periodontitis and gastrointestinal disease. This area of investigation, which is in its infancy, may represent another pathway for oral bacteria to cause systemic diseases and deserves more research.

Oral bacteria can spread through the body and have been associated with a variety of systemic diseases [1]. Thus, a report from Segata et al. [2] in Genome Biology found that oral cavity and stool bacteria overlapped in nearly half (45%) of the subjects in the Human Microbiome Project. Transfer of oral bacteria to the gut is therefore common. Members of the oral and oropharyngeal microbiota reach the stomach through swallowed saliva, nutrients, and drinks. Saliva production ranges from 0.75 to 1.5 L/day [3], but various stages of the disease can affect the production. The ingested saliva contains an enormous amount of oral bacteria. In general, these bacteria are poor colonizers of the healthy intestine [4]. In severe diseases, however, increased amounts of oral bacteria have been reported in the intestine, e.g. in inflammatory bowel disease, HIV infection, liver cirrhosis, colon cancer, primary sclerosing cholangitis, gastroesophageal reflux disease, and alcoholism (for a review see Atarashi et al. [5]). In patients with periodontitis, 10^8–^10^10^ of the keystone periodontal pathogen *Porphyromonas gingivalis* can be swallowed each day [6,7]. If oral bacteria can tolerate the harsh pH of the stomach, they may reside and proliferate in the gastrointestinal tract [8]. This is particularly the case with *P. gingivalis*, which is acid-resistant and may migrate to the colon and change colonic functions [8,9] (Figure 1). In support of this, *P. gingivalis* is thought to have a role in orodigestive cancers [10].

**Figure 1.**
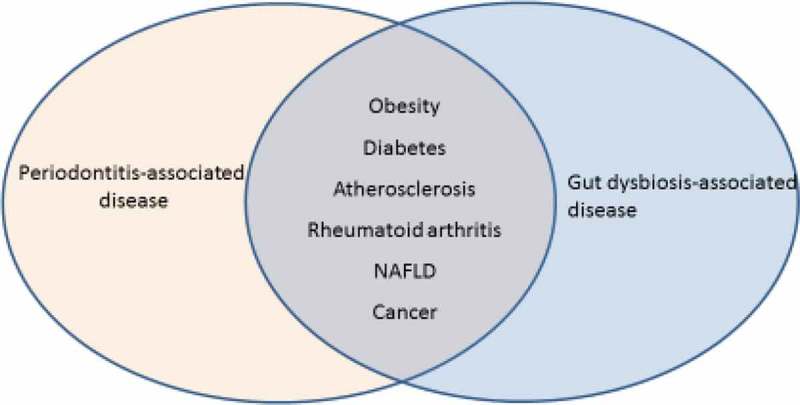
Diseases associated with periodontal disease are also associated with gut dysbiosis.

**Figure 2.**
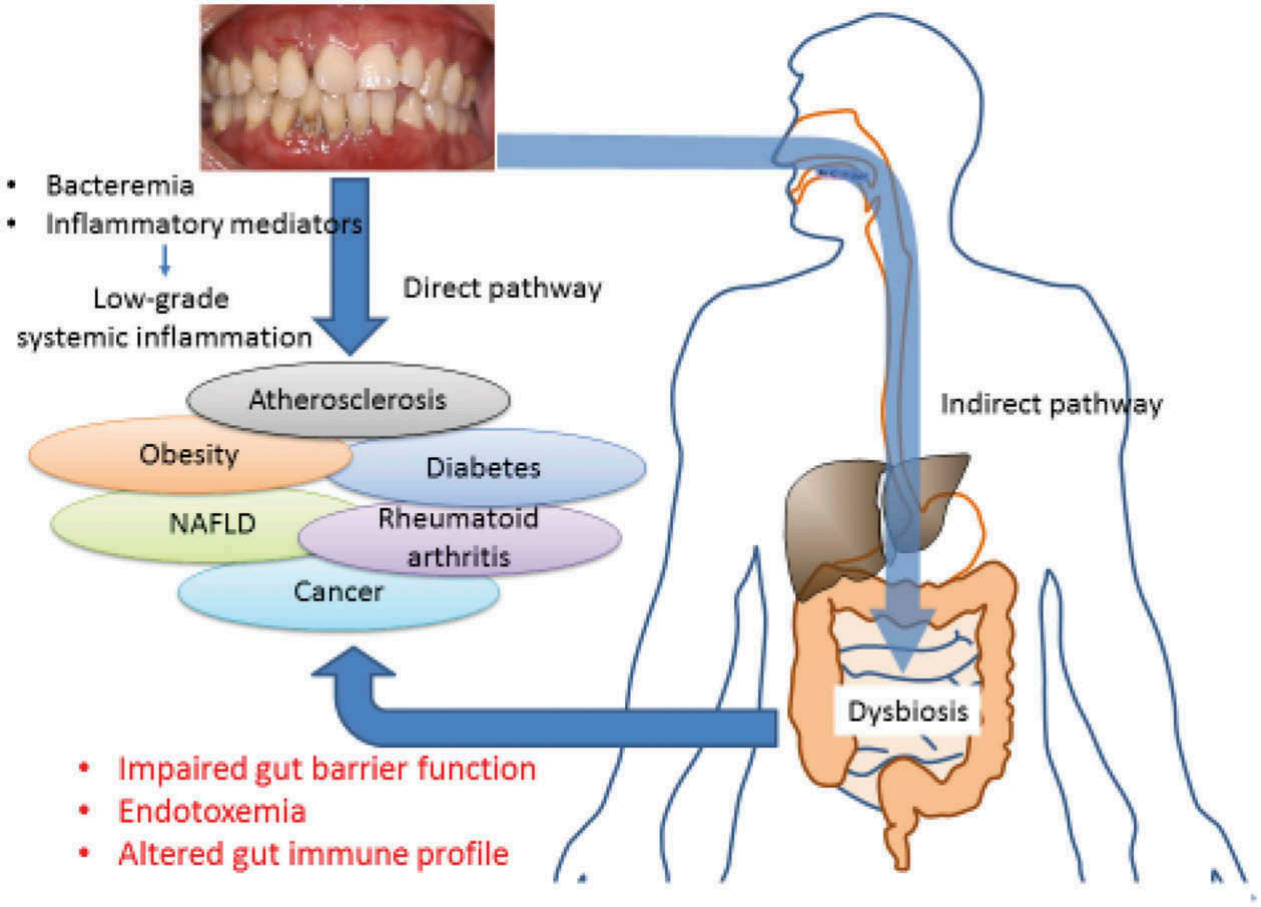
Possible mechanism for the link between periodontal disease and systemic diseases. A possible underlying mechanism is bacteremia/endotoxemia originating from periodontal pockets and systemic diffusion of inflammatory mediators from the lesion. The other mechanism is that impairment of the gut barrier function and modulation of the gut immune profile induced by dysbiotic oral bacteria-mediated gut dysbiosis result in endotoxemia and systemic inflammation.

*P. gingivalis* is a great manipulator of the oral microbiome and immune defense [11–14] and diseases related to periodontitis. It is often associated with dysbiosis in the gut microbiota [15–17]. The present Note discusses if oral bacteria, particularly *P. gingivalis*, can modulate the microbiome of the gut in animals and humans.

Disturbances in the gut microbiota by swallowed bacteria may lead to endotoxemia causing metabolic disorders (Figure 2). To test this, *P. gingivalis* was administered orally (2 times a week for 5 weeks) to mice (C57BL/6N). This caused increased levels of plasma endotoxin and insulin and reduced mRNA expression of the tight junction protein ZO-1 in the small intestine. Pyrosequencing showed that Bacteroidales was significantly increased [18]. *P. gingivalis* was not detected in the blood of the *P. gingivalis-*administered mice, but other bacterial DNA was found here. It was speculated that the endotoxemia was not necessarily induced by bacteria from the oral cavity but could be related to changes in the gut microbiota caused by oral bacteria that had been swallowed. If these results are confirmed, they suggest a new paradigm for the relationship between periodontitis and systemic diseases.

In another study with C57BL/6N mice, a single administration of *P. gingivalis* changed the gut microbiota significantly compared to sham-treated mice, with increased Bacteroidetes and decreased Firmicutes [11]. The mRNA expression of the tight junction proteins Tjp1 and Ocln, involved in intestinal permeability, was downregulated. Also, these changes were thought to be due to increases of endotoxin in the blood since there was no outgrowth of *P. gingivalis* in the gut. Disturbance in the composition of the gut microbiota occurred even after a single administration of *P. gingivalis*, linking periodontitis and systemic disease. The amount of *Porpyromonadaceae* in the fecal samples was less than 0.003%. This agreed with findings from another mouse model where *P. gingivalis* acted as a keystone pathogen in periodontitis disrupting host-microbial homeostasis when present at <0.01% of the total microbiota [19].

In 10 C57BL/6J mice, tooth ligatures imbibed with *P. gingivalis* were inserted to induce periodontitis, while 10 mice were sham-ligated [20]. 16S rRNA pyrosequencing detected no significant differences in the main phyla and in the genus *Parabacteroides* in the gut of the two groups, but the experimental group displayed significant alveolar bone loss. The ileum of the *P. gingivalis* group showed significantly upregulated occludin, claudin2, and NOD2. This suggested that periodontal inflammation, to some extent, had affected the mechanical and immune barrier functions of the mouse gut.

In a mouse (DBA/1J) experimental rheumatoid arthritis (RA) model, *P. gingivalis* was orally administered, intended to mimic periodontitis patients swallowing the bacterium. The gut microbiome changed, followed by increased serum endotoxin and inflammatory markers, and the gut barrier function was impaired [9]. A clear relationship between the oral and gut microbiota and RA was demonstrated. Thereafter, DBA/1J mice were given orally *P. gingivalis* and *Prevotella intermedia*, which contrary to *P. gingivalis* has no peptidyl arginine deiminase causing citrullination. This was followed by induction of collagen-induced arthritis (CIA). A significant change in the gut microbiome occurred [9]. *P. gingivalis* but not *P. intermedia* significantly increased IL-17 levels in sera and culture supernatants, as well as the Th17 cell proportions among mesenteric lymphocytes and Peyer’s patches. Although *P. gingivalis* aggravated CIA in the DBA/1J mice, it did not further increase the level of anti-citrullinated protein antibody. The suspected role of *P. gingivalis* in RA could, therefore, be by affecting the gut immune system and by causing composition shifts in the gut microbiota rather than by citrullination of proteins.

Periodontitis may affect non-alcoholic fatty liver disease (NAFLD) [18,21], the most common form of chronic liver disease. In addition, the gut microbiota has been suggested to mediate the development and progression of NAFLD [22–24]. C57BL/6J mice (8 weeks old) were given the periodontopathogen *Aggregatibacter actinomycetemcomitans* or saline for 6 weeks and either normal chow (NCAa, NCco) or high-fat diet (HFAa and HFco) [25]. The NCAa and HFAa groups represented mice with weakened glucose tolerance and insulin resistance compared to controls. Higher hepatic steatosis was detected in the HFAa mice than in the HFco animals after ingestion of *A. actinomycetemcomitans*, which affected NAFLD by changing the composition of the gut microbiota and glucose metabolism.

Infection with type II *P. gingivalis* in an NAFLD mouse model accelerated dramatically the progression of NAFLD [21], which was considerably faster than in control mice on a high-fat diet (HFD), but not those on a basal diet. HFD and *P. gingivalis* cooperatively increased the risk of NAFLD in this mouse model. Infection with other oral bacteria (*Streptococcus sanguinis* and *S. salivarius*) did not accelerate the progression of NAFLD, which might be due to a specific *P. gingivalis* effect under HFD conditions. The results should be interpreted with caution because the bacteria were administered via the jugular vein, not by the oral cavity. Furthermore, the control bacteria used in this study were inappropriate; future studies should use Gram-positive rods as controls for the test species (*P. gingivalis*) rather than Gram-positive cocci.

The effect of oral bacteria on the gut microbiota has also been studied in man. Several of these studies support findings from animal models. Thus, in patients with liver cirrhosis, a major change in the gut microbiota was due to the massive invasion of the gut by oral bacteria [26]. More than half (54%) of the patient-enriched, taxonomically assigned bacterial species were of oral origin (mostly veillonella and streptococci). This suggested that an invasion with mouth bacteria to the gut had taken place in the patients with liver cirrhosis. The correlation of the severity of liver cirrhosis with an abundance of the invading bacteria further indicated that oral bacteria other than *P. gingivalis* could also play a role in the pathology of liver cirrhosis.

*P. gingivalis* has been associated with NAFLD and non-alcoholic steatohepatitis (NASH) [21]. When *P. gingivalis* was examined from 150 biopsy-proven NAFLD patients (102 with non-alcoholic steatohepatitis, 48 with non-alcoholic fatty liver (NAFL), and 60 non-FADL control subjects), it was detected in the gut of the NAFLD patients at significantly higher levels than in non-NAFLD controls (46.7% vs 21.7%, odds ratio 3.16). This supported experiments in mice already mentioned [21]. Altogether, these animal and human studies indicate a clear association between *P. gingivalis* and NAFLD. Also in NASH, the more advanced stage of NAFLD, the detection frequency of *P. gingivalis* was higher than in non-NAFLD subjects (52.9%, odds ratio 3.91).

Komazaki et al. [25] examined the relationship between periodontitis and clinical/biochemical parameters in 52 NAFLD patients. IgG antibody titers were measured against *A. actinomycetemcomitans, P. gingivalis*, and *Fusobacterium nucleatum*. Only anti-*A. actinomycetemcomitans* antibody titers correlated positively with visceral fat, fasting plasma insulin and HOMA-IR (Homeostatic Model Assessment for Insulin Resistance), and negatively with the liver/spleen ratio.

When metagenomic shotgun sequencing and a metagenome-wide association study were made with dental, salivary, and fecal samples from a cohort of RA patients and healthy controls, concordance was detected between the oral and gut microbiomes. This suggested an overlap in the prevalence and function of bacterial species in the two body sites [27]. Alteration in the proportion of species was seen both in the oral and gut microbiota of the RA patients but was partially resolved after treatment of RA. The authors suggested that RA could represent a state of chronic inflammation that may be provoked or aggravated by the overgrowth of pathogenic bacteria or by the lack of immune modulating commensal bacteria.

In another study sequencing libraries of 16S rDNA were used to compare the subgingival biofilm and intestinal microbiota in patients (n = 40) with chronic periodontitis and metabolic syndrome, and healthy individuals (n = 40). *P. gingivalis, Tannerella forsythia*, and *Treponema denticola* appeared in high concentrations in the subgingival microbiota while the intestinal microbiome was dominated by *Enterobacteriaceae* and *Eubacteriaceae*. There were signs of intestinal dysbiosis, mostly related to a decrease of protective species [28]. Whether the oral bacteria were related to the intestinal dysbiosis was not examined.

Lourenço et al. [17] defined the gut microbiota in individuals with periodontal diseases. They found that individuals with periodontal diseases (gingivitis n = 14, “chronic” periodontitis n = 23), in contrast to animal models [11,18], had a less diverse intestinal microbiome. The gut microbiota was characterized by an increase in the Firmicutes/Bacteroides ratio and enrichment in Euryarcheota, Verrucomicrobiota, and Proteobacteria. Besides, high numbers of a large variety of oral taxa were associated with periodontal destruction and inflammation in the gut irrespective of the periodontal status. Several of them were genera of putative periodontal pathogens. How the oral microbiota survived in the mucosal barrier of the gut or circumvented it to establish an infection there was not considered.

The large amounts of swallowed dead bacteria from the mouth may stimulate several pathogens in the gut (necrotrophy) and create a new phenotype by upregulation of bacterial virulence genes (necrovirulence) and increased cytotoxicity. This has been demonstrated for periodontopathogens *in vitro* [29]. Cultures of *P. gingivalis* and *P. intermedia* exhibited a significant increase in growth when at least 10 dead bacterial cells were at disposal for one living cell. The growth of *P. intermedia* was stimulated much less by *Streptococcus oralis* and *Streptococcus gordonii* than by dead *P. gingivalis* and *P. intermedia*. Furthermore, gingipain virulence genes of *P. gingivalis* such as *rgpA, rgpB*, and *kgp* were upregulated in presence of dead *P. intermedia*. The long fimbriae gene *fimA* and the collagenase *prtC* gene were also upregulated.

There is an increasing amount of evidence suggesting that certain oral bacteria can contribute to oral and gastrointestinal cancers [10,30]. For example, *P. gingivalis* can be implicated in precancerous gastric and colon cancer lesions. Epidemiological studies have shown that there is an increased risk for such cancers in both men and women with periodontal disease or tooth loss [10]. *P. gingivalis* up-regulates specific receptors on oral squamous cell carcinoma cells and keratinocytes, induces epithelial-to-mesenchymal transition of oral epithelial cells, and activates metalloproteinase-9 and interleukin-8 in cultures of carcinoma cells. In addition, *P. gingivalis* accelerates cell cycling and suppresses apoptosis in cultures of oral epithelial cells [10]. There may be a direct relationship between *P. gingivalis* and orodigestive cancers, where the contribution to carcinogenesis may be due to the secondary intrusion of the oral bacterium outside of its primary location (oral cavity), yet still within anatomically continuous regions.

Association of oral bacteria with gastrointestinal cancer was also demonstrated by Flemer et al. [31] who profiled the microbiota in oral swabs and stools of patients with colorectal cancer (CRC) and control subjects by 16S rRNA gene sequencing. They found that several operational taxonomic units (OTUs) were shared between oral swabs and stool samples that may be potential tools for the detection of CRC. Some of these OTUs corresponded to late colonizers of oral biofilms such as *F. nucleatum, Parvimonas micra*, and *Peptostreptococcus stomatitis*. The oral microbiota in colorectal cancer was distinctive and predictive. Association of oral *F. nucleatum* with CRC was further confirmed by the isolation of an identical clone of *F. nucleatum* from the oral cavity and tissue specimens of CRC in the same subjects [32].

The effect of oral bacteria on the gut microbiota has also been demonstrated through favorable changes after periodontal therapy [33]. Thus, periodontal treatment in cirrhosis was associated with improvement in oral and gut dysbiosis, systemic inflammation, Model for End-Stage Liver Disease (MELD) score and cognitive function. The improvement was especially seen in patients with hepatic encephalopathy. Furthermore, non-surgical periodontal treatment for 3 months of 10 NAFLD patients reduced the serum levels of AST and ALT, which are sensitive indicators of liver damage or injury. Periodontal treatment may, therefore, be an efficient supportive measure in patients with NAFLD [21].

## Concluding remarks

The oral microbiome may have a great effect on the health of the gastrointestinal system. This has been reported in dental and medical journals of high impact. Both animal and human studies indicate that for example, *P. gingivalis* may influence the gut microbiota causing dysbiosis. This can happen despite the fact that the digestive tract hosts a much greater bacterial density than the oral cavity. Particularly long-term, orally ingested *P. gingivalis*, as seen in periodontitis, may affect intestinal dysbiosis. Also, the periodontopathogen *A. actinomycetemcomitans* may alter the gut microbiota but *P. gingivalis* and *A. actinomycetemcomitans* are not the only periodontopathogens that can translocate to extraoral sites. Actually, a large variety of oral species can reach the intestinal microbiota through swallowing, regardless of the periodontal status, but only a subset of these bacteria seems to colonize the gut when the microbiota here is dysbiotic. Whether this colonization requires a dysbiotic oral microbiota is not clear, but cannot be excluded. Anyhow, severe diseases and genetic susceptibility of the host may promote ectopic colonization of oral bacteria. Good oral hygiene, periodontal therapy, prebiotics, and probiotics may help ameliorating oral bacteria-elicited gastrointestinal disorders. The intestinal link may also be another pathway for oral bacteria to cause systemic inflammatory diseases. However, this area of research is still in its infancy and requires further investigation before firm conclusions can be drawn.
